# Supplementary material for: Fission yeast Dis1 is an unconventional TOG/XMAP215 that induces microtubule catastrophe to drive chromosome pulling
Source: Commun Biol. 2022 Nov 26;5:1298. doi: 10.1038/s42003-022-04271-2 (PMC9701203; doi:10.1038/s42003-022-04271-2)
Supplement: Supplementary file 1 — Supplementary Information [file 42003_2022_4271_MOESM1_ESM.pdf]

Supplementary Information

Fission yeast Dis1 is an unconventional TOG/XMAP215 that induces microtubule catastrophe to drive chromosome pulling

Yuichi Murase, Masahiko Yamagishi, Naoyuki Okada, Mika Toya, Junichiro Yajima, Takahiro Hamada and Masamitsu Sato

Supplementary Figures 1–5
Supplementary Tables 1–7
Supplementary References

Supplementary Figures

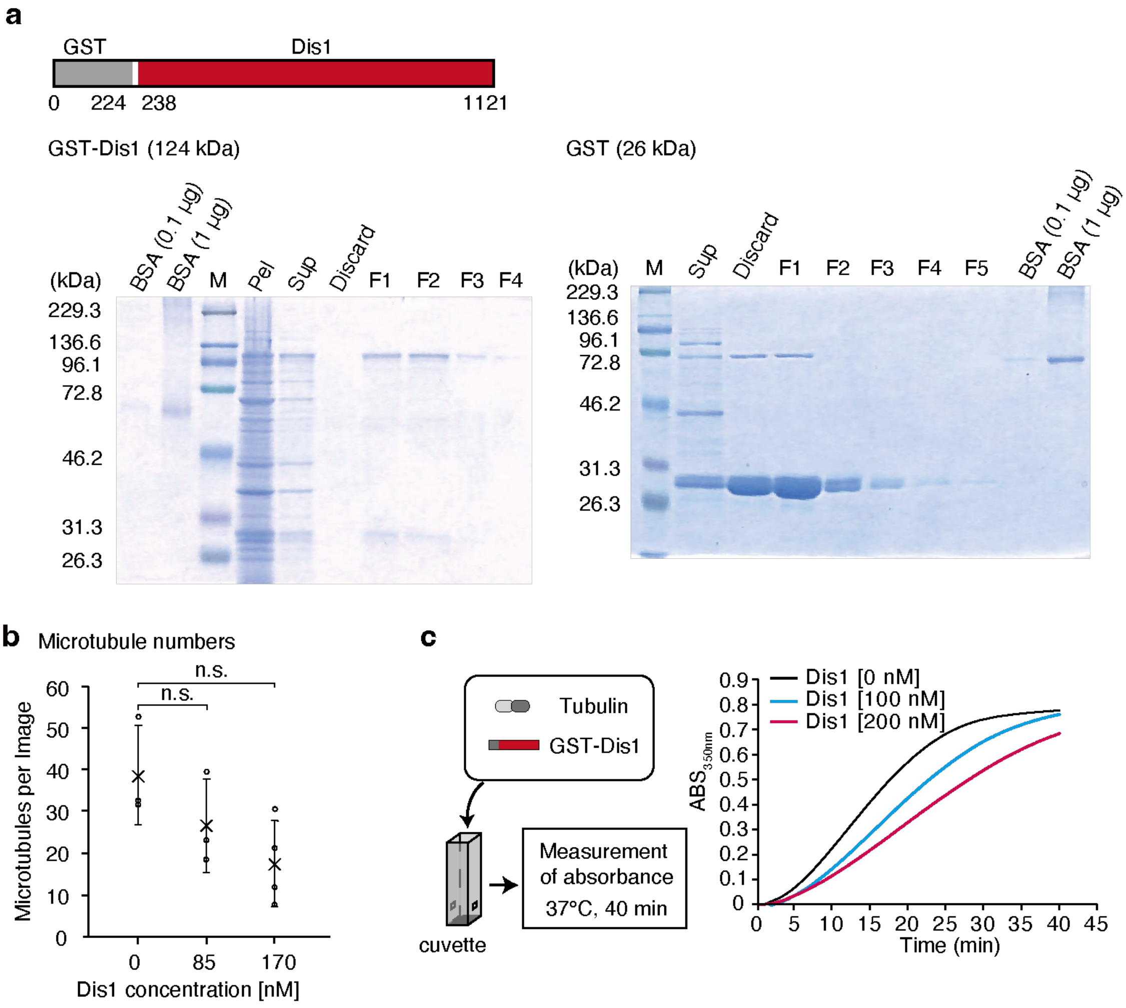

Supplementary Figure 1. GST-Dis1 and microtubule nucleation *in vitro*.

a A schematic for recombinant GST-Dis1 proteins used in this study. Purification of the recombinant GST-Dis1 from *E. coli* extract. Fractions of sequentially eluted samples for recombinant GST-Dis1 and GST (Discard, F1, F2, F3…) were analysed with SDS–PAGE followed by staining with Coomassie Brilliant Blue. Samples of the supernatant (Sup) and cell debris (Pel) after cell disruption were also applied. M, standards for molecular weights, shown on the left. **b** Average numbers of microtubules per observed field were plotted. The experimental procedures are as shown in **Fig. 1a**. Crosses, the mean; bullets, technical replicates (n > 3 for each concentration of Dis1). More than 4 fields were analysed in each replicate. Error bars are SD. The statistical significance of difference was determined using one-way ANOVA followed by Tukey–Kramer method. P values are shown; n.s., not significant. **c** Experimental outline for turbidity assays. Purified tubulin (26 µM) and 0–200 nM of GST-Dis1 were mixed and incubated at 37°C, and the absorbance (350 nm) was measured in 5-s intervals for 40 min. The kinetics of tubulin turbidity over time is shown as graphs. The representative of 4 technical repeats is shown.

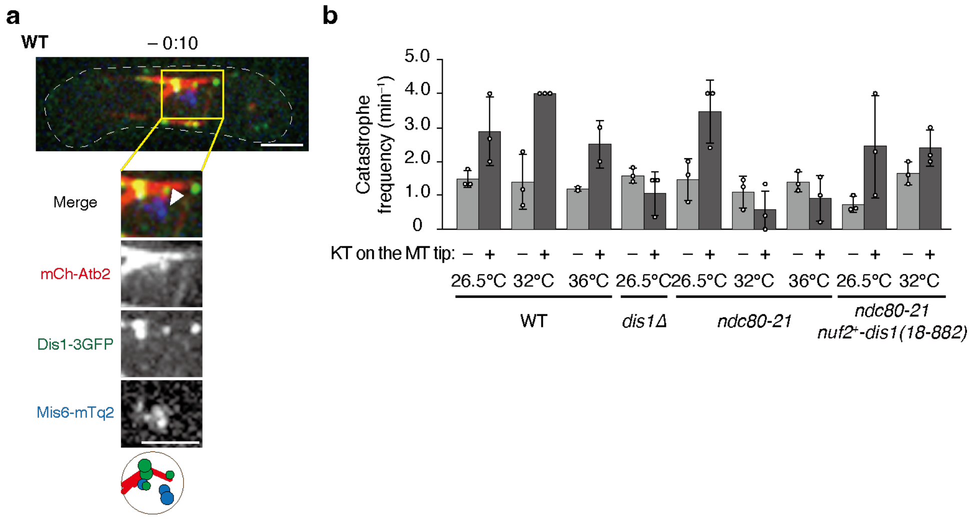

Supplementary Figure 2. Kinetochore retrievals via microtubule catastrophe in various strains.

a Images for a zygotic nucleus at the onset of meiosis I in a WT cell recorded 10 s before images of 0 min:00 s shown in **Fig. 2a**. Dis1 was not localised to KTs that were not bound to microtubules (arrowhead). Scale bar, 2 μm. **b** Catastrophe frequencies of kinetochore-microtubules in each strain under the indicated conditions. Meiosis was induced at 26.5°C and 32°C, similarly to **Fig. 2e**. Bullets represent 3 technical replicates, except for WT 36°C (2 replicates). Error bars, SD.

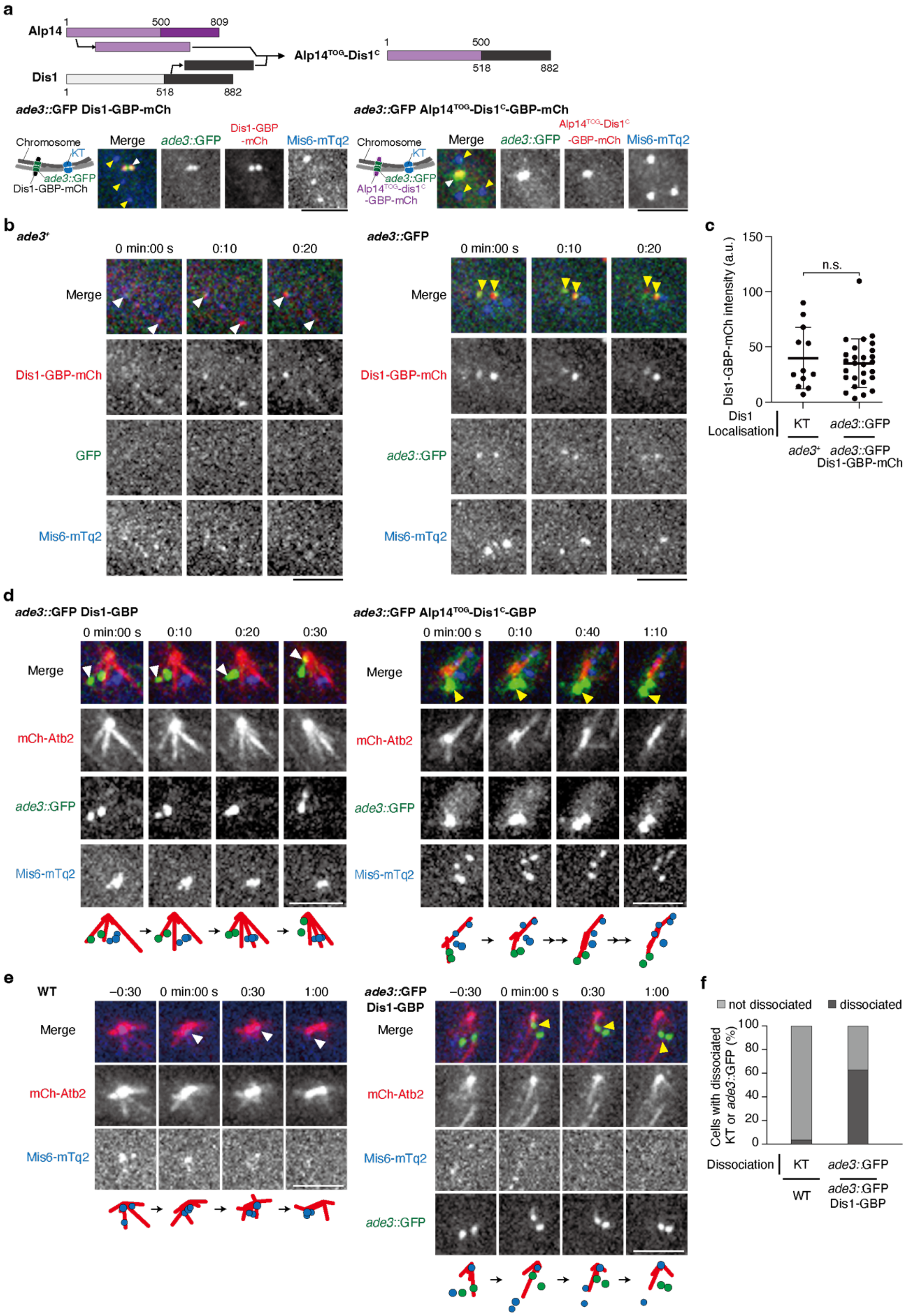
a
Alp14
1
500
809
Alp14TOG-Dis1C
1
500
518
882
Dis1
1
518
882
ade3::GFP Dis1-GBP-mCh
ade3::GFP Alp14TOG-Dis1C-GBP-mCh
Chromosome
KT
ade3::GFP
Dis1-GBP-mCh
Merge
ade3::GFP
Dis1-GBP
-mCh
Mis6-mTq2
Alp14TOG-dis1C
-GBP-mCh
Alp14TOG-Dis1C
-GBP-mCh
b
ade3+
ade3::GFP
0 min:00 s
0:10
0:20
Merge
Dis1-GBP-mCh
GFP
ade3::GFP
Mis6-mTq2
c
Dis1-GBP-mCh intensity (a.u.)
150
100
50
0
n.s.
Dis1
Localisation
KT
ade3::GFP
ade3+
ade3::GFP
Dis1-GBP-mCh
d
ade3::GFP Dis1-GBP
ade3::GFP Alp14TOG-Dis1C-GBP
0 min:00 s
0:10
0:20
0:30
0:40
1:10
Merge
mCh-Atb2
ade3::GFP
Mis6-mTq2
e
WT
ade3::GFP
Dis1-GBP
−0:30
0 min:00 s
0:30
1:00
Merge
mCh-Atb2
Mis6-mTq2
ade3::GFP
f
not dissociated
dissociated
Cells with dissociated
KT or ade3::GFP (%)
100
80
60
40
20
0
Dissociation
KT
ade3::GFP
WT
ade3::GFP
Dis1-GBP

Supplementary Figure 3. Retrieval of the *ade3::*GFP locus with Dis1-GBP or Alp14^{TOG}-Dis1^{C}-GBP by microtubules.

a A schematic for the chimeric protein Alp14^{TOG}-Dis1^{C} used in this study. The *ade3* locus located on a chromosome arm region was labelled with GFP by use of LacI-GFP/*LacO* system. Dis1-GBP-mCherry and Alp14^{TOG}-Dis1^{C}-GBP-mCherry, and the kinetochore marker Mis6-mTurquoise2 were visualised at room temperature. The GBP fusion proteins (Dis1-GBP and Alp14^{TOG}-Dis1^{C}-GBP) were colocalised with *ade3::*GFP (white arrowheads), validating that enforced oligomerisation of Dis1 (and Alp14^{TOG}-Dis1^{C}) at the *ade3::*GFP loci was successful. Note that neither Dis1-GBP-mCherry nor Alp14^{TOG}-Dis1^{C}-mCherry predominantly localised to kinetochores (marked by Mis6-mTurquoise2, yellow arrowheads). **b,c** Comparison of the Dis1 amount at kinetochores and at the *ade3::*GFP locus. In *ade3*$^{+}$ (WT) cells without GFP expression, Dis1-GBP-mCherry accumulated at kinetochores (white arrowheads, **b**). In *ade3::*GFP cells, Dis1-GBP-mCherry predominantly accumulated at *ade3::*GFP (yellow arrowheads). Signal intensities of Dis1-GBP-mCherry at kinetochores (KT, **c**) and at *ade3::*GFP were plotted. Bold lines, means. n = 12 (KT) and 27 (*ade3::*GFP) foci of Dis1-GBP-mCherry. Error bars, SD. n.s., not significant (Student's two tailed t-test). **d** Time-lapse images for nuclei of *ade3::*GFP Dis1-GBP and *ade3::*GFP Alp14^{TOG}-Dis1^{C}-GBP cells at the onset of meiosis I filmed together with mCherry-Atb2 (microtubules) and Mis6-mTurquiose2 (kinetochores). The *ade3* locus was retrieved by microtubules in cells expressing Dis1-GBP (white arrowheads), but was frequently unretrieved in cells expressing the chimera-GBP (yellow arrowheads). These images were processed for kymographs shown in **Fig. 4a**. **e** Retention of retrieved chromosomes at spindle poles was monitored in WT and *ade3::*GFP Dis1-GBP nuclei at room temperature. Kinetochores (Mis6-mTurquoise2) were retrieved by microtubules and retained around SPBs (white arrowheads) in WT cells, whereas the *ade3::*GFP locus was frequently dissociated from SPBs (yellow arrowheads) even once retrieved by microtubules. Scale bars, 3 µm. **f** The percentage of WT cells (n = 28 cells) in which kinetochores were once retrieved to SPBs or the spindle, but dissociated from there. The percentage of *ade3::*GFP Dis1-GBP cells (n = 27 cells) in which the *ade3::*GFP loci was once retrieved but dissociated. The tendency of *ade3::*GFP dissociation was statistically confirmed by the $\chi 2$ two-sample test ($\chi 2 = 22$, $P < 0.005$).

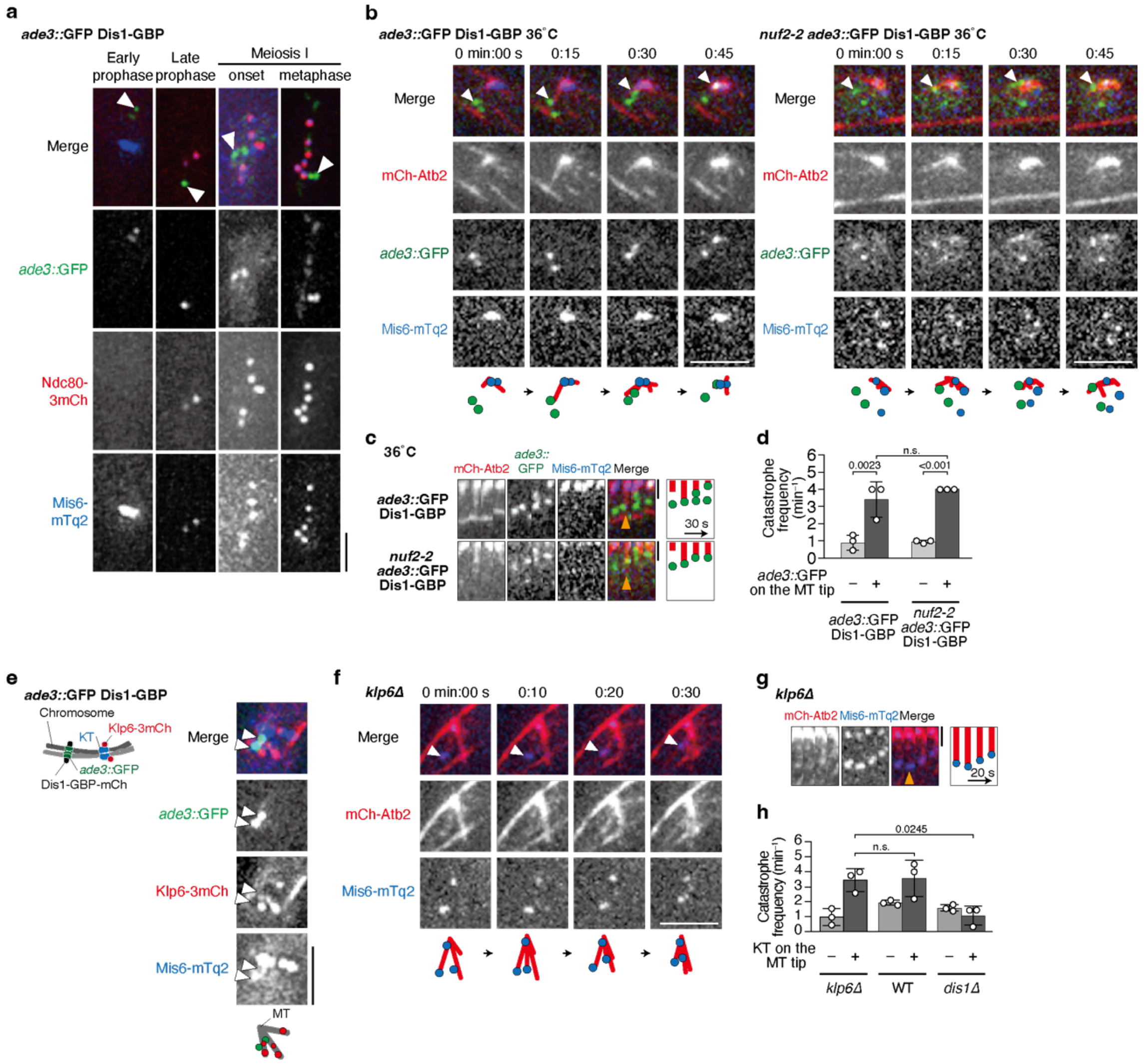

Supplementary Figure 4. Neither the Ndc80 complex nor Klp6 participates in microtubule shrinkage.

a Images of nuclei of *ade3::*GFP Dis1-GBP cells at the indicated stages of meiosis at room temperature. Ndc80-3mCherry localised nowhere (Early prophase) but to kinetochores in late prophase onwards (Mis6-mTurquoise2) [1,2]. Note that Ndc80-3mCherry was not recruited to *ade3::*GFP loci at any stage (arrowheads). Brightness and contrast of the Ndc80-3mCherry images were enhanced to clearly judge whether it localises to the *ade3::*GFP site. **b,c** Time-lapse images of a nucleus at the onset of meiosis I in *ade3::*GFP Dis1-GBP and *nuf2-2 ade3::*GFP Dis1-GBP cells at 36°C filmed together with mCherry-Atb2 (microtubules) and Mis6-mTurquiose2 (kinetochores). In both cells, the *ade3* locus was retrieved by microtubules (arrowheads). Kymographs of the microtubules with *ade3::*GFP are also shown (**c**). The orange arrowhead indicates start of microtubule catastrophe. **d** Catastrophe frequencies of microtubules with and without *ade3::*GFP at their tips were measured for *ade3::*GFP Dis1-GBP and *nuf2-2 ade3::*GFP Dis1-GBP cells. Bullets, technical replicates: n = 3 for each of four cases. **e** A meiotic nucleus with *ade3::*GFP loci expressing Dis1-GBP was filmed together with Klp6-3mCherry and kinetochores

(Mis6-mTurquoise2) at room temperature. Klp6-3mCherry did not co-localise with *ade3::*GFP at the onset of meiosis I (arrowheads). **f,g** Time-lapse images of a nucleus of the *klp6*Δ mutant at the onset of meiosis I at room temperature. Microtubules (mCherry-Atb2) and kinetochore (Mis6-mTurquoise2) were visualised. A kinetochore retrieved by a microtubule is marked with a white arrowhead, **f**. A kymograph for the event is shown (**g**). The orange arrowhead pinpoints the start of catastrophe. Scale bars, 3 µm (**a,b,e,f**); 1 µm (**c,g**). **h** Catastrophe frequencies of microtubules with or without kinetochores in *klp6*Δ cells. Bullets, technical replicates: n = 3 (+KT *klp6*Δ), 3 (–KT *klp6*Δ). The data for WT and *dis1*Δ cells are reprise of those in **Fig. 4** shown as a reference**.** Error bars, SD. The statistical significance of difference was determined using one-way ANOVA followed by Tukey–Kramer method. P values are shown; n.s., not significant.

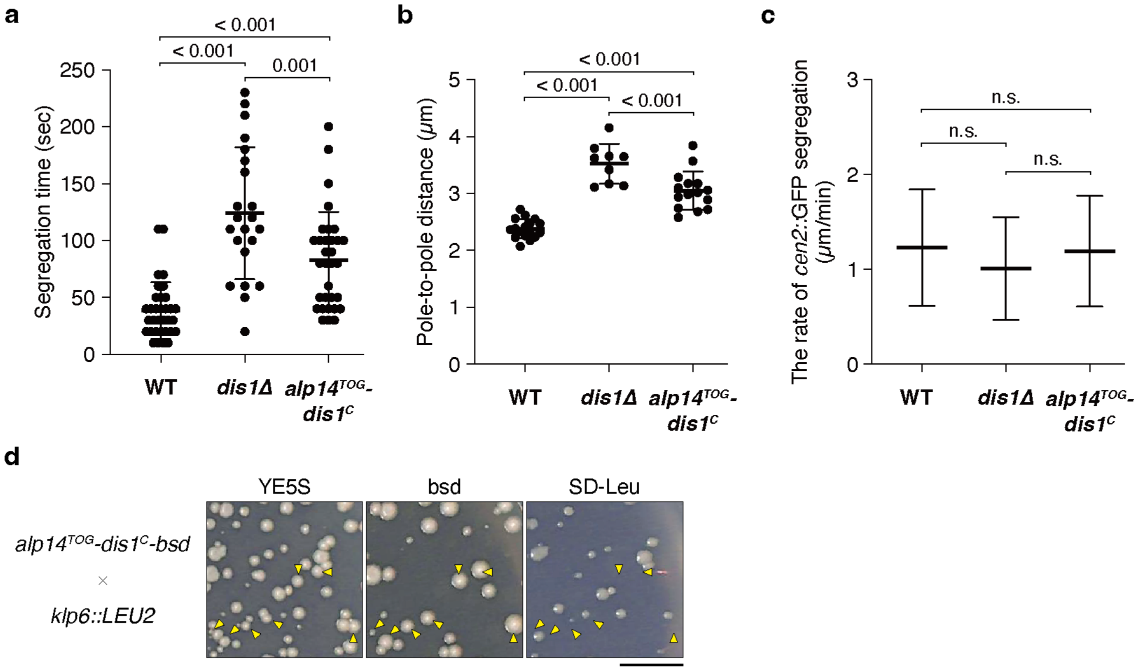

Supplementary Figure 5. Involvement of Dis1 in spindle microtubules during anaphase A of mitosis.

a Duration from separation of *cen2::*GFP signals at the anaphase A onset until reach to SPBs. n = 33 (WT), 22 (*dis1*Δ), 32 (*alp14^{TOG}-dis1^{C}*) *cen2::*GFP. **b** The pole-to-pole distance at the timing when *cen2::*GFP signals separated at the onset of anaphase A in the indicated strains. n = 17 (WT), 9 (*dis1*Δ), 15 (*alp14^{TOG}-dis1^{C}*) cells. **c** Relative velocities of separating *cen2::*GFP dots to SPBs during anaphase A in the indicated strains. n = 49 (WT), 59 (*dis1*Δ), 88 (*alp14^{TOG}-dis1^{C}*) depolymerisation events. Anaphase A cells analysed herein are identical to those shown in **Fig. 5**. Error bars; SD. The statistical significance of difference was determined using one-way ANOVA followed by Tukey–Kramer method. P values are shown; n.s., not significant. **d** Spores from a genetic crossing of *alp14^{TOG}-dis1^{C}-bsd* and *klp6::LEU2* mutants were directly spread onto a non-selective YE5S plate for germination followed by colony formation (30°C). The colonies were then replica-plated onto YE5S plates with or without Blasticidin S (bsd) or SD–Leu plate (SD media lacking leucine) and incubated at 30°C. Colonies conferred blasticidin S resistance (arrowheads) lacked leucine autotrophy without exception, indicating that the double mutant was inviable. The *klp6* gene is located on the chromosome II, whereas *alp14^{TOG}-dis1^{C}* has been inserted to the *dis1* locus on chromosome III, therefore these two genes are not genetically linked. Scale bar, 1 cm.

Supplementary Tables

Supplementary Table 1. Parameters of microtubule dynamics *in vitro*

Dis1 concentration [nM]	The number of microtubules (sum)	Catastrophe frequency (min^{-1})	Rescue frequency (min^{-1})	Growth rate ($\mu m \cdot min^{-1}$)	Shrinkage rate ($\mu m \cdot min^{-1}$)
0	95	0.0728 ± 0.0196	0.100 ± 0.200	0.978 ± 0.189	22.5 ± 4.59
50	124	0.111 ± 0.0221	0.803 ± 0.255	1.19 ± 0.0809	19.8 ± 1.98
100	148	0.114 ± 0.0102	1.46 ± 0.656	1.20 ± 0.112	17.4 ± 2.96
200	162	0.118 ± 0.0175	1.52 ± 0.564	1.44 ± 0.130	15.4 ± 3.88

Frequencies of catastrophe and rescue, and rates for growth and shrinkage are mean ± SD (*n* = 4 experiments). See methods for details.

Supplementary Table 2. Parameters of microtubule dynamics in wild-type and *dis1*Δ cells

Genotype	Existence of Dis1 and KT on the microtubule tip	The total number of microtubules (sum)	Catastrophe frequency (min^{-1})
WT	–Dis1–KT	22	1.7 ± 0.36
WT	+Dis1–KT	17	2.4 ± 0.20
WT	+Dis1+KT	11	3.6 ± 1.2
*dis1*Δ	*dis1*Δ –KT	52	1.6 ± 0.22
*dis1*Δ	*dis1*Δ +KT	15	1.1 ± 0.64

Catastrophe frequencies of microtubules at meiosis I onset in strains of each genotype were calculated. Observed microtubules were classified depending on whether each microtubule displayed Dis1 and kinetochores (KT) on the tip. The total number of microtubules observed in 3 experiments are also shown. The frequencies are mean ± SD (3 experiments).

Supplementary Table 3. Parameters of microtubule dynamics in wild-type, *ndc80-21* and *ndc80-21 nuf2⁺-dis1(18-882)* cells

Genotype	Existence of KT on the microtubule tip	Temperature	The total number of microtubules (sum)	Catastrophe frequency (min^{-1})
WT	–KT	26.5°C	22	1.5 ± 0.26
WT	+KT	26.5°C	9	2.9 ± 1.0
WT	–KT	32°C	16	1.4 ± 0.81
WT	+KT	32°C	8	4.0 ± 0.00
WT	–KT	36°C	7	1.2 ± 0.062
WT	+KT	36°C	6	2.5 ± 0.71
ndc80-21	–KT	26.5°C	9	1.5 ± 0.61
ndc80-21	+KT	26.5°C	7	3.5 ± 0.92
ndc80-21	–KT	32°C	18	1.1 ± 0.46
ndc80-21	+KT	32°C	17	0.58 ± 0.68
ndc80-21	–KT	36°C	8	1.4 ± 0.29
ndc80-21	+KT	36°C	10	0.92 ± 0.67
ndc80-21 nuf2⁺-dis1(18-882)	–KT	26.5°C	15	0.73 ± 0.23
ndc80-21 nuf2⁺-dis1(18-882)	+KT	26.5°C	9	2.4 ± 1.5
ndc80-21 nuf2⁺-dis1(18-882)	–KT	32°C	11	1.6 ± 0.34
ndc80-21 nuf2⁺-dis1(18-882)	+KT	32°C	11	2.4 ± 0.53

Catastrophe frequencies of microtubules at meiosis I onset were calculated for each strain with the indicate genotype at the indicated temperature. Observed microtubules were classified depending on whether each microtubule displayed kinetochores (KT) on the tip. The total number of microtubules observed in 2 experiments (WT, 36°C) or 3 experiments (others) are also shown. The frequencies are mean ± SD (2 experiments for WT, 36°C; 3 experiments for others).

Supplementary Table 4. Parameters of microtubule dynamics in *ade3::*GFP Dis1-GBP and *ade3::*GFP Alp14^{TOG}-Dis1^{C}-GBP cells

Genotype	Existence of *ade3::*GFP on the microtubule tip	The total number of microtubules (sum)	Catastrophe frequency (min^{-1})
*ade3::*GFP Dis1-GBP	–*ade3::*GFP	30	1.3 ± 0.027
*ade3::*GFP Dis1-GBP	+*ade3::*GFP	14	2.3 ± 0.29
*ade3::*GFP Alp14^{TOG}-Dis1^{C}-GBP	–*ade3::*GFP	26	1.3 ± 0.33
*ade3::*GFP Alp14^{TOG}-Dis1^{C}-GBP	+*ade3::*GFP	17	0.98 ± 0.12

Catastrophe frequencies of microtubules at meiosis I onset in strains of each genotype were calculated. Observed microtubules were classified depending on whether each microtubule displayed *ade3::*GFP foci on the tip. The total number of microtubules observed in 3 experiments are also shown. The frequencies are mean ± SD (3 experiments).

Supplementary Table 5. Parameters of microtubule dynamics in *ade3::*GFP Dis1-GBP and *nuf2-2 ade3::*GFP Alp14^{TOG}-Dis1^{C}-GBP cells

Genotype	Existence of *ade3::*GFP on the microtubule tip	The total number of microtubules (sum)	Catastrophe frequency (min^{-1})
*ade3::*GFP Dis1-GBP	–*ade3::*GFP	9	0.89 ± 0.44
*ade3::*GFP Dis1-GBP	+*ade3::*GFP	14	3.4 ± 1.0
nuf2-2 *ade3::*GFP Dis1-GBP	–*ade3::*GFP	6	0.95 ± 0.091
nuf2-2 *ade3::*GFP Dis1-GBP	+*ade3::*GFP	9	4.0 ± 0.00

Catastrophe frequencies of microtubules at meiosis I onset in strains of each genotype were calculated. Observed microtubules were classified depending on whether each microtubule displayed *ade3::*GFP foci on the tip. The total number of microtubules observed in 3 experiments are also shown. The frequencies are mean ± SD (3 experiments).

Supplementary Table 6. Parameters of microtubule dynamics in *klp6*Δ cells

Genotype	Existence of KT on the microtubule tip	The total number of microtubules (sum)	Catastrophe frequency (min^{-1})
*klp6*Δ	–KT	32	0.97 ± 0.55
*klp6*Δ	+KT	14	3.4 ± 0.76

Catastrophe frequencies of microtubules at meiosis I onset in *klp6*Δ cells were calculated. Observed microtubules were classified depending on whether each microtubule displayed Dis1 and kinetochores (KT) on the tip. The total number of microtubules observed in 3 experiments are also shown. The frequencies are mean ± SD (3 experiments).

Supplementary Table 7. *S. pombe* strains used in this study

Strain	Alias	Genotype	Origin	Related figures
YM0439	Wild type	*h90 dis1-3GFP-kan Z2-mCherry-atb2-hph mis6-mTurquoise2-nat leu1 ura4 ade6-M216*	This study	Figure 2a, 2b, 2c, 3a, 3b, 3c, 4c, 4d, S2a, S3e, S3f
YM0451	*dis1Δ*	*h90 dis1::ura4+ Z2-mCherry-atb2-hph mis6-mTurquoise2-nat leu1 ura4 ade6-M216*	This study	Figure 2a, 2b, 2c, 4d
YM0455	Wild type	*h90 Z2-mCherry-atb2-hph mis6-mTurquoise2-nat leu1 ura4 ade6-M216*	This study	2d, 2e, S2b
YM0512	*ndc80-21*	*h90 ndc80-21-kan Z2-mCherry-atb2-hph mis6-mTurquoise2-nat leu1 ura4 ade6-M216*	This study	Figure 2d, 2e, S2b
YM0540	*ndc80-21 nuf2^{+}-dis1(18-882)*	*h90 ndc80-21-bsd nuf2+-dis1(18-882)-ura4+ Z2-mCherry-atb2-hph mis6-mTurquoise2-nat leu1 ura4 ade6-M216*	This study	Figure 2d, 2e, S2b
YM0567	*ade3::GFP dis1-GBP*	*h90 dis1-GBP-bsd ade3::LacO-ura4+-kan his7+-LacI-GFP Z2-mCherry-atb2-hph mis6-mTurquoise2-nat leu1 ura4 ade6-M216*	This study	Figure 4a, 4b, 4c, 4d, S3d, S3e, S3f, S4b, S4c, S4d
YM0604	*ade3::GFP*	*h90 dis1::alp14^{TOG}-dis1^{C}-GBP-bsd*	This study	Figure 4a,

	alp14^{TOG}-dis1^{C}-GBP	*ade3::LacO-ura4+-kan his7+-LacI-GFP Z2-mCherry-atb2-hph mis6-mTurquoise2-nat leu1 ura4 ade6-M216*		4b, 4d, S3d
MJ0018	Wild type	*h90 sfi1-mRFP-hph cen2-LacO-kan-ura4+ his7+-LacI-GFP leu1 ura4 lys1 ade6-M216*	Our stock	Figure 5, S5a, S5b, S5c
RD0025	*dis1Δ*	*h90 dis1::bsd sfi1-mRFP-hph cen2-LacO-kan-ura4+ his7+-LacI-GFP leu1 ura4 ade6-M216*	Our stock	Figure 5, S5a, S5b, S5c
YM0918	*alp14^{TOG}-dis1^{C}*	*h90 dis1::alp14^{TOG}-dis1^{C} sfi1-mRFP-hph cen2-LacO-kan-ura4+ his7+-LacI-GFP leu1 ura4 ade6-M216*	This study	Figure 5, S5a, S5b, S5c
YM0586	*ade3::GFP dis1-GBP-mCh*	*h90 dis1-GBP-mCherry-hph ade3::LacO-ura4+-kan his7+-LacI-GFP mis6-mTurquoise2-nat leu1 ura4 ade6-M216*	This study	Figure S3a, S3b, S3c
YM0620	*ade3::GFP alp14^{TOG}-dis1^{C}-GBP-mCh*	*h90 dis1::alp14^{TOG}-dis1^{C}-GBP-mCherry-hph ade3::LacO-ura4+-kan his7+-LacI-GFP mis6-mTurquoise2-nat leu1 ura4 ade6-M216*	This study	Figure S3a
YM0924	Wild type	*h90 dis1-GBP-mCherry-hph mis6-mTurquoise2-nat leu1 ura4 ade6-M216*	This study	Figure S3b, S3c
YM0634	*ade3::GFP dis1-GBP ndc80-3mCh*	*h90 ndc80-3mCherry-hph dis1-GBP-bsd ade3::LacO-ura4+-kan his7+-LacI-GFP mis6-mTurquoise2-nat leu1 ura4 ade6-M216*	This study	Figure S4a
YM0932	*nuf2-2 ade3::GFP Dis1-GBP*	*h90 nuf2-2::ura4+ dis1-GBP-bsd ade3::LacO-ura4+-kan his7+-LacI-GFP mis6-mTurquoise2-nat leu1 ura4 ade6-M216*	This study	Figure S4b, S4c, S4d
YM0635	*ade3::GFP dis1-GBP klp6-3mCh*	*h90 klp6-3mCherry-hph dis1-GBP-bsd ade3::LacO-ura4+-kan his7+-LacI-GFP mis6-mTurquoise2-nat leu1 ura4 ade6-M216*	This study	Figure S4e
YM0925	*klp6Δ*	*h90 klp6::LEU2 Z2-mCherry-atb2-hph mis6-mTurquoise2-nat leu1 ura4*	This study	Figure S4f, S4g, S4h

YM0936	*alp14^{TOG}-dis1^{C}-bsd*	*h90 alp14^{TOG}-dis1^{C}-bsd leu1 ura4*	This study	Figure S5d
LJ0272	*klp6Δ*	*h90 klp6::LEU2 leu1 ura4*	Our stock	Figure S5d

Supplementary References

1. Nabetani, A., Koujin, T., Tsutsumi, C., Haraguchi, T. & Hiraoka, Y. A conserved protein, Nuf2, is implicated in connecting the centromere to the spindle during chromosome segregation: a link between the kinetochore function and the spindle checkpoint. *Chromosoma* **110**, 322–334 (2001).

2. Asakawa, H., Hayashi, A., Haraguchi, T. & Hiraoka, Y. Dissociation of the Nuf2-Ndc80 Complex Releases Centromeres from the Spindle-Pole Body during Meiotic Prophase in Fission Yeast. *Mol. Biol. Cell* **16**, 2325–2338 (2005).
