# Supplementary material for: Fission yeast Dis1 is an unconventional TOG/XMAP215 that induces microtubule catastrophe to drive chromosome pulling
Source: Commun Biol. 2022 Nov 26;5:1298. doi: 10.1038/s42003-022-04271-2 (PMC9701203; doi:10.1038/s42003-022-04271-2)
Supplement: Supplementary file 2 — Description of Additional Supplementary Files [file 42003_2022_4271_MOESM2_ESM.pdf]

Description of Additional Supplementary Files

File name: Supplementary Data

Description: Source data underlying the graphs presented in the main and supplementary figures.
