# Supplementary material for: Fission yeast Dis1 is an unconventional TOG/XMAP215 that induces microtubule catastrophe to drive chromosome pulling
Source: Commun Biol. 2022 Nov 26;5:1298. doi: 10.1038/s42003-022-04271-2 (PMC9701203; doi:10.1038/s42003-022-04271-2)
Supplement: Supplementary file 4 — Reporting Summary [file 42003_2022_4271_MOESM4_ESM.pdf]

nature portfolio

Corresponding author(s): MASAMITSU SATO

Last updated by author(s): Nov 9, 2022

Reporting Summary

Nature Portfolio wishes to improve the reproducibility of the work that we publish. This form provides structure for consistency and transparency in reporting. For further information on Nature Portfolio policies, see our Editorial Policies and the Editorial Policy Checklist.

Statistics

For all statistical analyses, confirm that the following items are present in the figure legend, table legend, main text, or Methods section.

n/a	Confirmed	
☐	☒	The exact sample size (*n*) for each experimental group/condition, given as a discrete number and unit of measurement
☐	☒	A statement on whether measurements were taken from distinct samples or whether the same sample was measured repeatedly
☐	☒	The statistical test(s) used AND whether they are one- or two-sided<br>*Only common tests should be described solely by name; describe more complex techniques in the Methods section.*
☒	☐	A description of all covariates tested
☐	☒	A description of any assumptions or corrections, such as tests of normality and adjustment for multiple comparisons
☐	☒	A full description of the statistical parameters including central tendency (e.g. means) or other basic estimates (e.g. regression coefficient) AND variation (e.g. standard deviation) or associated estimates of uncertainty (e.g. confidence intervals)
☐	☒	For null hypothesis testing, the test statistic (e.g. *F*, *t*, *r*) with confidence intervals, effect sizes, degrees of freedom and *P* value noted<br>*Give P values as exact values whenever suitable.*
☒	☐	For Bayesian analysis, information on the choice of priors and Markov chain Monte Carlo settings
☒	☐	For hierarchical and complex designs, identification of the appropriate level for tests and full reporting of outcomes
☒	☐	Estimates of effect sizes (e.g. Cohen's *d*, Pearson's *r*), indicating how they were calculated

Our web collection on statistics for biologists contains articles on many of the points above.

Software and code

Policy information about availability of computer code

Data collection	SoftWoRx v3.7.0 and v.6.5.1 (Applied Precision) for image acquisition in microscopy of cells, IQ3 (Andor) for image acquisition in microscopy of microtubules in vitro, as described in the method section of the manuscript.
Data analysis	Images of microtubules in vitro were analysed using Fiji v2.0.0-rc-69/1.52q (National Institutes of Health), and images of microtubules in meiocytes and mitotic cells were analysed using SoftWoRx v3.7.0 and v.6.5.1 (Applied Precision), Illustlator (Adobe) and Fiji. Statistical analysis were performed by software R v3.6.2 and Microsoft Excel, as described in the method section of the manuscript.

For manuscripts utilizing custom algorithms or software that are central to the research but not yet described in published literature, software must be made available to editors and reviewers. We strongly encourage code deposition in a community repository (e.g. GitHub). See the Nature Portfolio guidelines for submitting code & software for further information.

Data

Policy information about availability of data

All manuscripts must include a data availability statement. This statement should provide the following information, where applicable:

- Accession codes, unique identifiers, or web links for publicly available datasets
- A description of any restrictions on data availability
- For clinical datasets or third party data, please ensure that the statement adheres to our policy

The data presented in this study are available from the corresponding author upon reasonable request.

Human research participants

Policy information about studies involving human research participants and Sex and Gender in Research.

Reporting on sex and gender	This report did not contains human researches.
Population characteristics	This report did not contains human researches.
Recruitment	This report did not contains human researches.
Ethics oversight	This report did not contains human researches.

Note that full information on the approval of the study protocol must also be provided in the manuscript.

Field-specific reporting

Please select the one below that is the best fit for your research. If you are not sure, read the appropriate sections before making your selection.

☒ Life sciences ☐ Behavioural & social sciences ☐ Ecological, evolutionary & environmental sciences

For a reference copy of the document with all sections, see nature.com/documents/nr-reporting-summary-flat.pdf

Life sciences study design

All studies must disclose on these points even when the disclosure is negative.

Sample size	For the results of measuring microtubule dynamics in vitro and of analyzing kinetochore pulling in cells, the sample size was sufficient, showing the same trend as when the results of all experiments were combined (data not shown).<br>Experiments measuring the fluorescence of Dis1-3GFP at microtubule ends were n>14. The sample size is sufficient as a small size shows the same trend (data not shown).<br>The number of microtubules classified for kinetochore and ade3::GFP recovery is n>8. The sample size is sufficient as this result shows the same trend as the result for catastrophe frequency.
Data exclusions	In the in vitro assay, microtubule speckle was observed and microtubules were not included in the sample if they slid significantly. For meiocyte observations, the spatial arrangement of microtubules was examined by analysing the microtubule image stacks, and data where microtubule ends were not yeilded the observed space were omitted.
Replication	For measuring microtubule dynamics in in vitro assays, N = 4 independent experiments were done. For measuring microtubule length in in vitro assays, N = 3 independent experiments were done.<br>When representative images are shown, at least two repeats were performed.<br>For experiments measuring microtubule dynamics in cells, there are N=3 experiments, except for WT 36°C. For WT 36°C, there are N=2.<br>For the Turbidity assay, 6 experiments were repeated; the same trend was confirmed in 4 experiments of the 6 results.<br>The experiment to measure the amount of Dis1-GBP-mCh accumulated on ade3::GFP was repeated twice.
Randomization	All microtubules in the in vitro assay and all radial microtubules in meiocytes were analysed. For microscopy observation and quantification, cells were randomly chosen.
Blinding	All analyses were monitored by at least two people.

Reporting for specific materials, systems and methods

We require information from authors about some types of materials, experimental systems and methods used in many studies. Here, indicate whether each material, system or method listed is relevant to your study. If you are not sure if a list item applies to your research, read the appropriate section before selecting a response.

Materials & experimental systems

n/a	Involved in the study
☒	☐ Antibodies
☒	☐ Eukaryotic cell lines
☒	☐ Palaeontology and archaeology
☒	☐ Animals and other organisms
☒	☐ Clinical data
☒	☐ Dual use research of concern

Methods

n/a	Involved in the study
☒	☐ ChIP-seq
☒	☐ Flow cytometry
☒	☐ MRI-based neuroimaging
